# CRISPR/Cas9-Mediated Editing of *Bsr-d1* and *Pi21* Enhances Blast Resistance in a High-Quality Rice Maintainer Line

**DOI:** 10.3390/plants15172585

**Published:** 2026-08-25

**Authors:** Ke Lan, Lin Yuan, Dacheng Zhao, Xixi Ma, Jinlian Yang, Hu Wu, Fang Liu, Shengwu Chen, Rongbai Li

**Affiliations:** 1 State Key Laboratory for Conservation and Utilization of Subtropical Agro-Bioresources, College of Agriculture, Guangxi University, Nanning 530004, China; lankke1@163.com (K.L.);; 2 Guangxi Lvhai Seed Co., Ltd., Nanning 530007, China

**Keywords:** rice, rice blast, CRISPR/Cas9, *Bsr-d1*, *Pi21*

## Abstract

Rice (*Oryza sativa* L.) is a staple food crop worldwide, and improving disease resistance is a core target in rice breeding. In this study, we employed CRISPR/Cas9 genome editing to modify the coding sequence (CDS) of two susceptibility genes, *Bsr-d1* and *Pi21*, in the elite maintainer line Gengxiang B to enhance its blast resistance. We generated *Bsr-d1/Pi21* double homozygous mutants via *Agrobacterium*-mediated genetic transformation. Quantitative RT-PCR revealed significantly suppressed transcript accumulation of both target genes in the edited lines compared with the wild type Gengxiang B. Upon inoculation with *Magnaporthe oryzae*, multiple defense-related marker genes were markedly upregulated in the double mutants. Phenotypic assays demonstrated significantly reduced disease severity for both leaf and panicle blast in the edited lines compared with the wild type. Importantly, no statistically detectable differences were found between the double mutants and wild-type plants for key agronomic or grain quality traits. Collectively, these results demonstrate that CRISPR/Cas9-mediated editing of susceptibility loci generates genetically stable blast-resistant rice germplasm without compromising agronomic traits or grain quality, providing valuable genetic resources for future rice varietal improvement.

## 1. Introduction

Rice (*Oryza sativa* L.) sustains more than half of the global human population, making its stable production critical to national food security and social stability. Rice blast, caused by the ascomycete fungus *Magnaporthe oryzae* (syn. *Pyricularia oryzae*), ranks among the most destructive biotic constraints in rice cultivation and is widely described as the “cancer” of rice. The pathogen infects rice at all developmental stages and all plant organs, with leaf blast and panicle neck blast inflicting the most severe yield losses. During severe epidemic outbreaks, complete crop failure may occur, and global annual yield reductions attributable to blast range from 10% to 30%, posing severe threats to food supply security [1]. *M. oryzae* exhibits extensive genetic diversity, rapid virulence mutation rates, complex infection cycles, and strong environmental adaptability, rendering conventional disease control extremely challenging [2].

For decades, blast management has relied heavily on synthetic fungicides. However, chemical control escalates production costs, contaminates agricultural ecosystems, and drives the emergence of fungicide-resistant pathogen populations. Therefore, breeding and deploying disease-resistant rice varieties is universally recognized as the most cost-effective, sustainable, and eco-friendly disease management strategy [3]. Traditional resistance breeding primarily depends on the discovery and introgression of major resistance (R) genes. Most R genes encode nucleotide-binding site–leucine-rich repeat (NBS-LRR) proteins, which trigger host hypersensitive cell death by recognizing race-specific avirulence effectors secreted by pathogens, conferring race-exclusive qualitative resistance [4]. Molecular marker-assisted selection (MAS) [5,6] has greatly accelerated the pyramiding and field deployment of broad-spectrum R genes such as *Pi9* and *Pigm*, facilitating the development of multiple resistant breeding lines [7,8]. Nevertheless, R gene-mediated immunity follows the canonical gene-for-gene interaction model [9,10]. Under persistent selective pressure imposed by pathogen populations, novel virulent races readily overcome single R gene resistance, leading to rapid breakdown of field resistance [11,12]. Accordingly, developing rice varieties with durable, broad-spectrum blast resistance has become a central research priority in crop disease resistance genetic improvement.

To circumvent the limitations of single R gene deployment, modern resistance improvement strategies have diverged into two complementary research avenues: (1) pyramiding multiple R genes with distinct resistance spectra to broaden immunity coverage; and (2) targeted editing of host susceptibility (S) genes to reinforce basal plant immunity at the molecular level [13]. The MAS-based R gene pyramiding approach has achieved notable breeding success [7,8], yet stacked R genes remain vulnerable to novel virulent pathogen races, and many favorable resistance alleles suffer from unfavorable linkage drag. In contrast, S gene knockout presents a transformative alternative: inactivating host factors that facilitate pathogen colonization or repress endogenous plant immunity delivers wider-spectrum, potentially longer-lasting basal resistance [14]. The rapid maturation and widespread adoption of CRISPR/Cas9 genome editing technology have drastically accelerated progress in this S gene engineering pipeline.

CRISPR/Cas9 technology has fundamentally reshaped crop functional genomics and precision breeding owing to its high efficiency, precision, and ease of operation [15]. It has been widely applied in rice blast resistance improvement in the following areas: (1) knockout of S genes, for example, successful editing of *OsERF922* significantly enhanced blast resistance by relieving its suppression of defense responses [16], and editing of S genes such as *OsDjA2* and *OsERF104* further confirmed the universality of this strategy [17]; (2) creation or simulation of broad-spectrum resistance alleles, for instance, functional disruption of *Bsr-d1* through editing recapitulated its natural broad-spectrum resistance allele and achieved rapid introduction of durable resistance [18,19]; and (3) multi-gene synchronous editing and trait pyramiding, as demonstrated by editing multiple S genes, including *OsERF922* and *OsSEC3A*, which produced stronger and more stable resistance than single-gene editing [14]. Editing multiple *OsS5H* genes also confirmed this synergistic effect [20]. Furthermore, combining disease resistance gene editing with fragrance, male sterility, and high yield has demonstrated the great potential of multi-trait integrated improvement [21,22]. These advances indicate that rice disease resistance breeding has entered a new era of rationally designed “genome editing breeding”.

Among the characterized blast resistance regulators, *Bsr-d1* and *Pi21* represent two functionally well-characterized S genes with substantial translational breeding value. *Bsr-d1* encodes a C_2_H_2_ zinc-finger transcription factor that negatively regulates blast resistance by suppressing peroxidase-mediated defense pathways. Natural loss-of-function alleles of *Bsr-d1* relieve this suppression, leading to enhanced H_2_O_2_ accumulation and broad-spectrum, durable blast resistance [18,19]. Importantly, *Bsr-d1* has been widely introgressed into elite rice backgrounds without significant adverse effects on major agronomic traits, further supporting its breeding utility [18,19]. *Pi21* encodes a proline-rich protein that functions as a negative regulator of basal defense against blast. Recessive loss-of-function haplotypes of *Pi21* release the suppression of basal defense responses, resulting in field-effective durable blast resistance [23,24,25]. Similar to *Bsr-d1*, the *pi21* allele has been successfully deployed in breeding programs without obvious yield penalties [23,24]. Although both genes have been individually characterized and utilized, direct molecular interactions between *Bsr-d1* and *Pi21* have not been reported to date, and their potential synergistic regulatory effect on blast resistance remains largely unknown. In the present study, distinct target sites within the coding sequences of *Bsr-d1* and *Pi21* were selected, and to our knowledge, the specific editing sites used here differ from those reported in previous studies. This provides novel allelic variants for functional characterization and breeding applications.

Conventional sexual crossing encounters persistent bottlenecks when stacking favorable S gene alleles, including severe linkage drag and limited diversity of available germplasm. By assembling a multi-guide RNA (gRNA) CRISPR/Cas9 construct, we simultaneously edited *Bsr-d1* and *Pi21* to enhance blast resistance while preserving the superior agronomic performance of elite recipient lines. Here, we delivered CRISPR/Cas9-mediated edits to the coding sequences of *Bsr-d1* and *Pi21* in Gengxiang B, an elite high-quality indica rice maintainer line. Our objectives were to generate stable blast-resistant edited germplasm via targeted sequence modification and supply superior genetic materials for hybrid rice varietal development.

## 2. Results

### 2.1. Generation of Bsr-d1 and Pi21 Mutant Lines via CRISPR/Cas9 Editing

**Figure 1:**
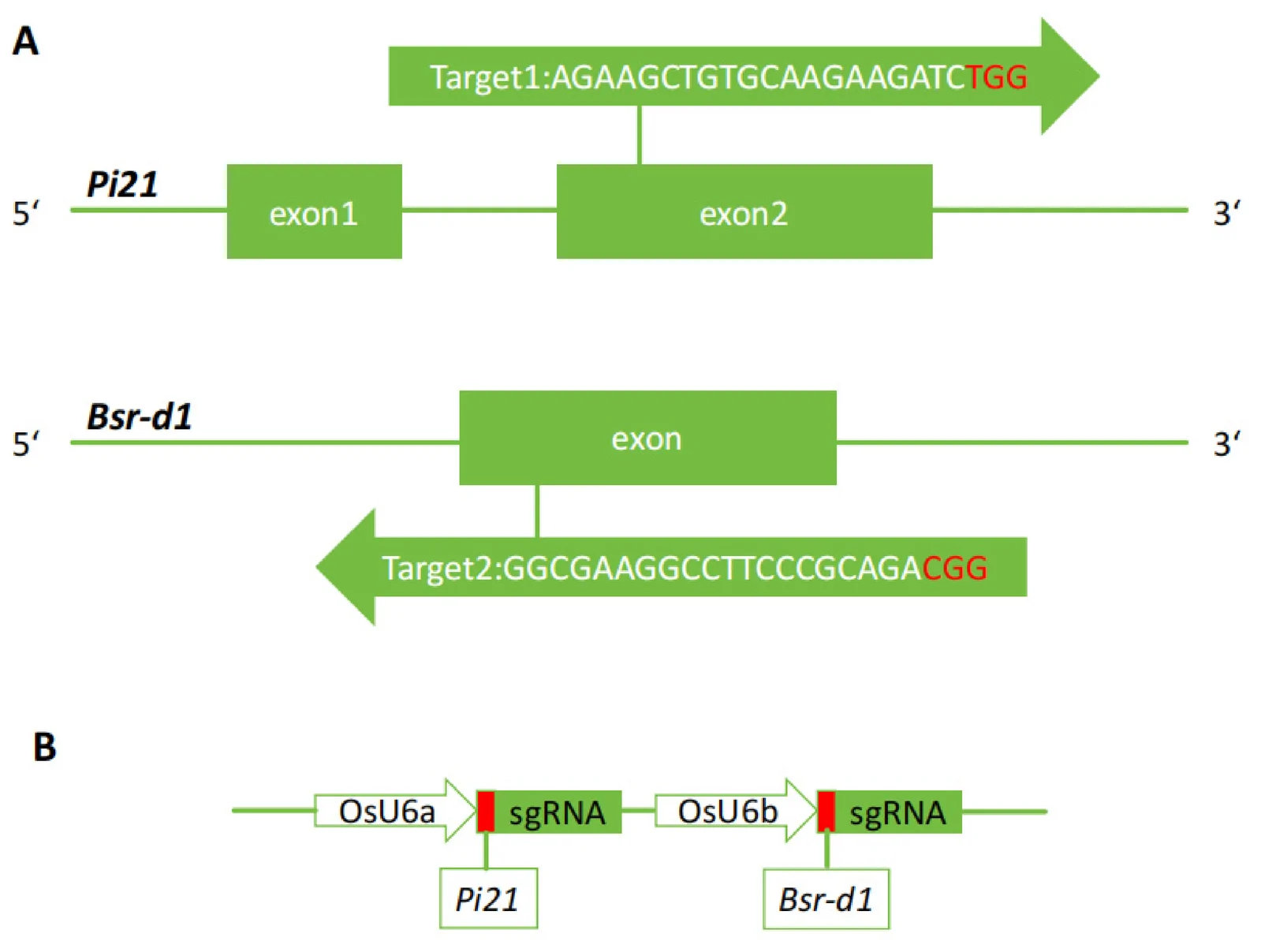
CRISPR/Cas9 target design for *Bsr-d1* and *Pi21*. (**A**) Gene structure of *Pi21* (2 exons) and *Bsr-d1* (1 exon). The target sequence of *Pi21* (Target1: AGAAGCTGTGCAAGAAGATCTG) is located in the second exon, and the target sequence of *Bsr-d1* (Target2: GGCAAGGCCCTTCCCGCAGCGG) is located in its single exon. Arrows indicate the 5′→3′ orientation; the three red bases within the target sequences represent the PAM. (**B**) Promoter–sgRNA expression cassette. The OsU6a and OsU6b promoters drive sgRNAs targeting *Pi21* and *Bsr-d1*, respectively. Red regions indicate the target sites; the arrow is for illustration only.

To obtain *Bsr-d1* (LOC_Os03g32230) and *Pi21* (LOC_Os04g32850) knockout mutants, two sgRNAs targeting the CDS region of *Bsr-d1* and *Pi21* were designed (Figure 1A) and ligated into the CRISPR/Cas9 vector (Figure 1B). The constructed vector was introduced into Gengxiang B via *Agrobacterium tumefaciens* EHA105, and 15 positive transgenic plants were obtained. Among these 15 transgenic seedlings, 14 carried mutations at the *Bsr-d1* target site, corresponding to a mutation rate of 93.33%, while only one plant remained unedited. Of the 14 *Bsr-d1*-edited plants, 11 were homozygous and 3 were heterozygous, giving homozygous and heterozygous mutation rates of 73.33% and 20.00%, respectively. At the *Pi21* target site, 10 of the 15 transgenic plants were mutated, corresponding to a mutation rate of 66.67%. Of these, 9 were homozygous and 1 was heterozygous, giving homozygous and heterozygous mutation rates of 60.00% and 6.67%, respectively. Among the 15 transgenic plants, 8 were homozygous double-gene mutants, corresponding to a double-gene homozygous mutation rate of 53.33% (Table 1). The mutation types at the *Bsr-d1* locus included insertions, substitutions and deletions, with frequencies of 46.67%, 6.67% and 40.00%, respectively, whereas only deletions were detected at the *Pi21* locus, with a frequency of 66.67% (Table 2).

Because CRISPR/Cas9 editing may cause off-target effects, potential off-target sites were analyzed in silico using the CRISPR-GE tool. No significant high-risk off-target sites were detected across the rice genome, and primers were designed to examine the predicted target regions (Table 3).

### 2.2. Bsr-d1/Pi21 Double Mutants Exhibit Markedly Enhanced Blast Resistance

**Figure 2:**
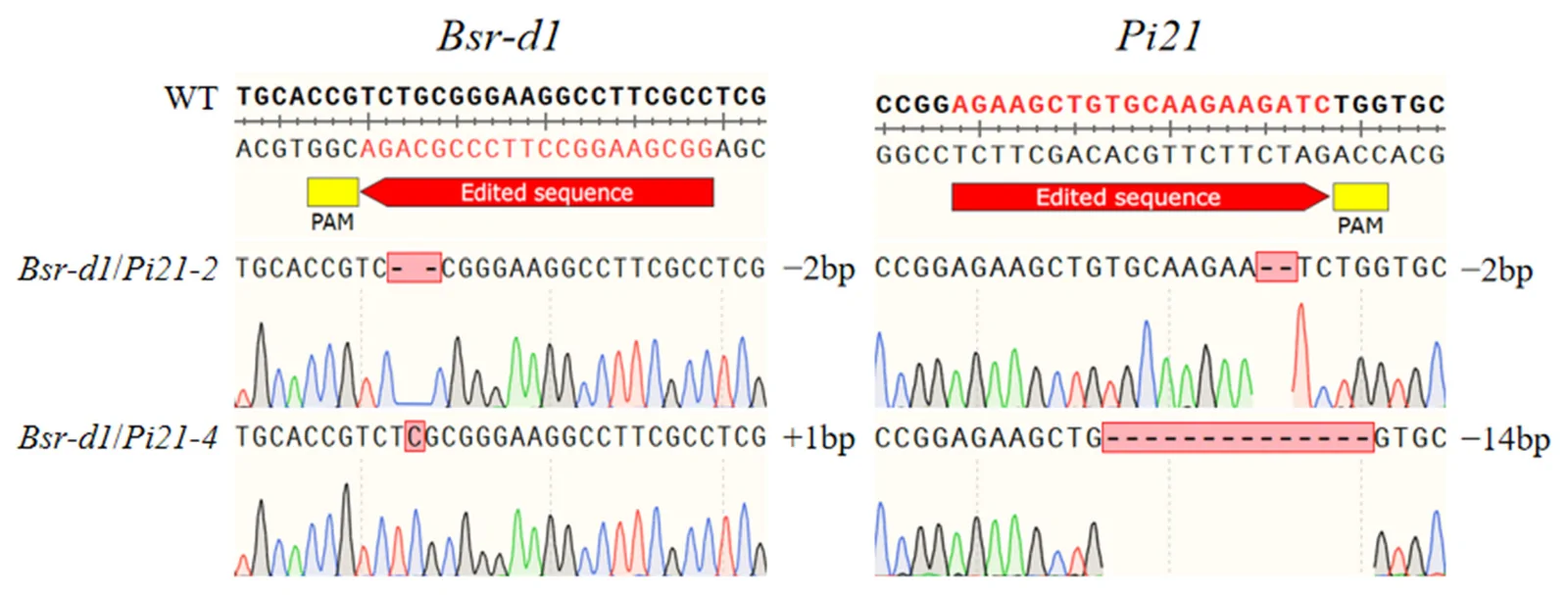
Different mutation types of *Bsr-d1* and *Pi21* in double-mutant plants. Sequence changes at the *Bsr-d1* and *Pi21* target sites. The sizes of deletions and insertions are indicated by a minus sign (−) and a plus sign (+), respectively. Red arrows indicate the 5′→3′ orientation, and yellow boxes indicate the PAM sequences.

Two homozygous double mutant lines with different mutation types, *Bsr-d1*/*Pi21*-2 and *Bsr-d1*/*Pi21*-4, were selected from the T_0_ generation for rice blast resistance evaluation (Table 4). *Bsr-d1*/*Pi21*-2 carries a 2 bp deletion at the *Bsr-d1* locus and a 2 bp deletion at the *Pi21* locus. *Bsr-d1*/*Pi21*-4 carries a 1 bp insertion at the *Bsr-d1* locus and a 14 bp deletion at the *Pi21* locus. The 14 bp deletion is expected to cause a frameshift mutation (Figure 2).

**Figure 3:**
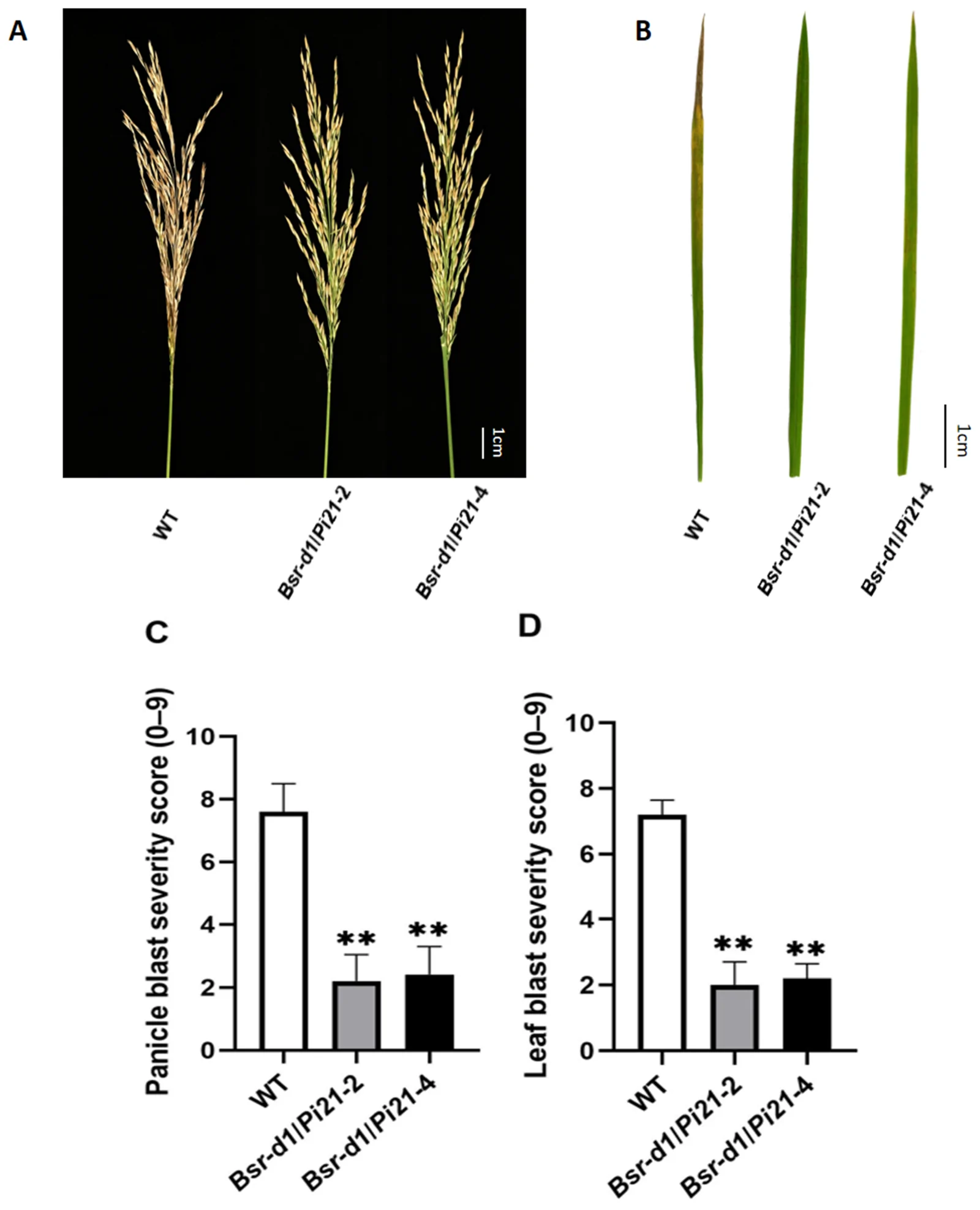
Comparison of disease phenotypes of rice panicle blast and leaf blast in *Bsr-d1/Pi21* double mutant lines with the wild type. (**A**) Representative panicle neck blast symptoms. (**B**) Representative leaf blast symptoms at 7 dpi. (**C**) Panicle blast severity score (0–9). (**D**) Leaf blast severity score (0–9). The wild-type (WT) plants showed severe sensitivity to *M. oryzae* infection, whereas the two double mutants (*Bsr-d1*/*Pi21*-2 and *Bsr-d1*/*Pi21*-4) exhibited markedly reduced disease symptoms and significantly lower severity scores. Data are mean ± SD (*n* = 5 panicles or leaves per line). ** *p* < 0.01 by one-way ANOVA followed by Dunnett’s test.

To evaluate blast resistance, *Bsr-d1*/*Pi21*-2, *Bsr-d1*/*Pi21*-4 and the wild-type control were inoculated with *Magnaporthe oryzae* isolate H322 by spray inoculation at the three-leaf stage. Leaf blast severity was scored at 7 days post-inoculation (dpi) to assess resistance at the seedling stage. In addition, the two double mutants and wild-type plants were grown in a natural rice blast nursery, and panicle blast severity was evaluated at the panicle stage. Quantitative disease evaluation based on a 0–9 severity scale showed that the wild type had a mean leaf blast severity score of 7.2 ± 0.4, whereas *Bsr-d1*/*Pi21*-2 and *Bsr-d1*/*Pi21*-4 had scores of 2.0 ± 0.7 and 2.2 ± 0.4, respectively (*p* < 0.01, one-way ANOVA followed by Dunnett’s test; Figure 3C, Table S2). Similarly, the mean panicle blast severity score of the wild type was 7.6 ± 0.9, whereas the two double mutants had scores of 2.2 ± 0.8 and 2.4 ± 0.9, respectively (*p* < 0.01, one-way ANOVA followed by Dunnett’s test; Figure 3D, Table S2). The corresponding disease index values are also presented in Table S2.

### 2.3. Transcript Profiling of Bsr-d1, Pi21, and Defense Regulatory Genes

**Figure 4:**
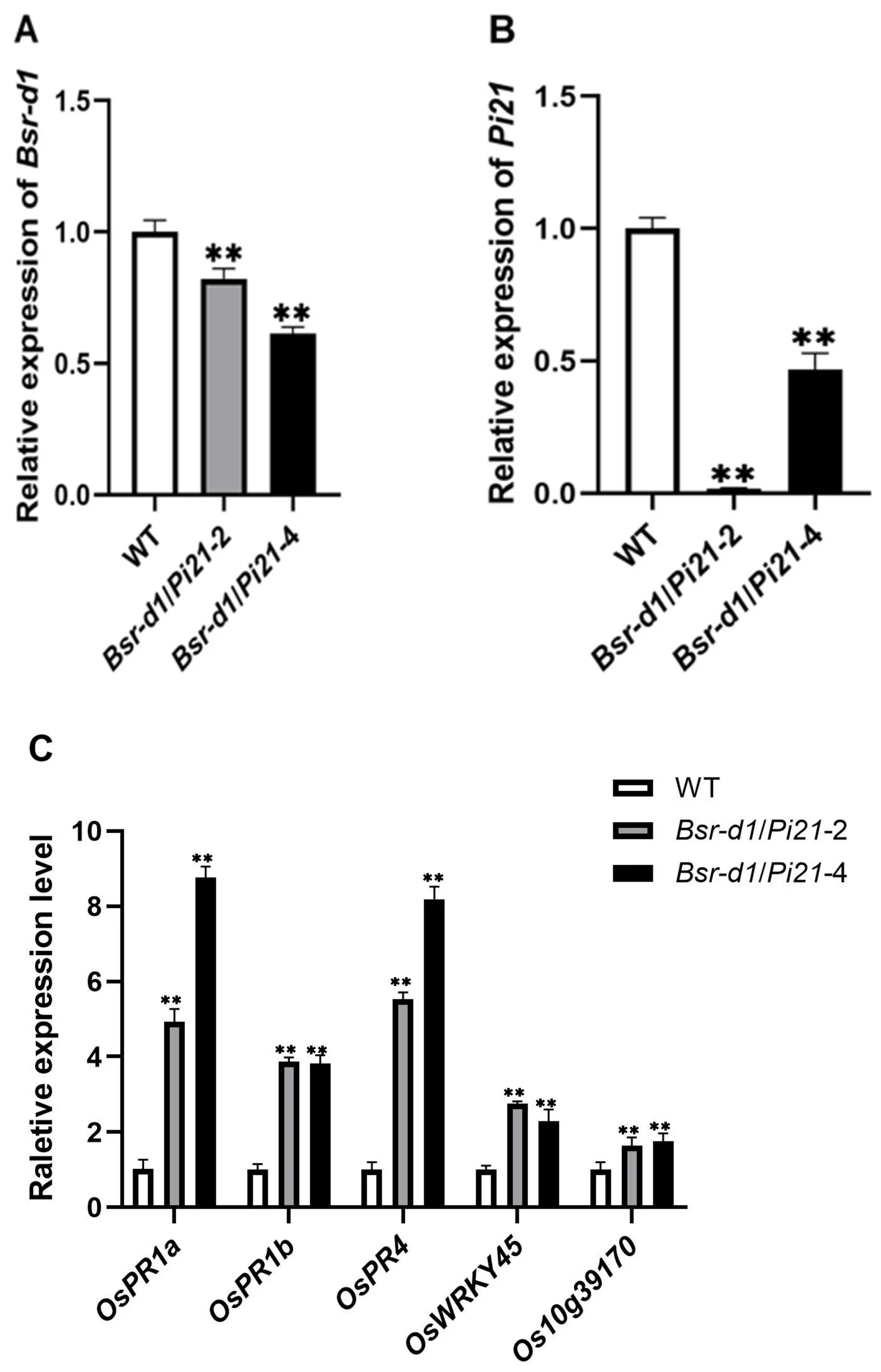
Expression analysis of target genes and defense-related genes in wild-type (WT) and *Bsr-d1*/*Pi21* double mutants. (**A**) Relative expression of *Bsr-d1*. (**B**) Relative expression of *Pi21*. (**C**) Relative expression of defense-related genes after *M. oryzae* infection. Data are mean ± SD (*n* = 3). ** *p* < 0.01 by one-way ANOVA followed by Dunnett’s test.

*Bsr-d1* and *Pi21* genes are rice blast susceptibility genes that facilitate disease development. Their expression levels are closely related to disease severity [18,19,23,24,25]. To further investigate the roles of *Bsr-d1* and *Pi21* in rice blast resistance, we compared their relative expression levels between the double mutants and wild-type Gengxiang B by qRT-PCR, using *OsActin* as the internal control. qRT-PCR analysis revealed that the transcript levels of both *Bsr-d1* and *Pi21* were significantly lower in the *Bsr-d1*/*Pi21* double mutants than in the wild type (*p* < 0.01, one-way ANOVA followed by Dunnett’s test; Figure 4A,B). This confirms that CRISPR/Cas9 editing effectively reduced the accumulation of both target gene transcripts, which is consistent with the enhanced blast resistance observed in the double mutants.

Upon pathogen infection, immune responses are activated through hormone signaling pathways [19,23]. To investigate the roles of *Bsr-d1* and *Pi21* in susceptibility pathways, leaves were collected from inoculated plants at 24 h post-inoculation, and the expression of the salicylic acid (SA) pathway marker genes *OsPR1a*, *OsPR1b*, and *OsWRKY45*, the jasmonic acid (JA) pathway marker gene *OsPR4*, and the peroxidase pathway marker gene *Os10g39170* was examined. All tested defense-related genes were significantly up-regulated in the double mutants compared with the wild type (*p* < 0.05, one-way ANOVA followed by Dunnett’s test) (Figure 4C), indicating that simultaneous knockout of *Bsr-d1* and *Pi21* not only down-regulated susceptibility gene expression but also activated defense-related pathways, promoted defense gene expression, and ultimately enhanced rice blast resistance.

### 2.4. Major Agronomic and Grain Quality Traits Are Unaltered in Double Mutants

**Figure 5:**
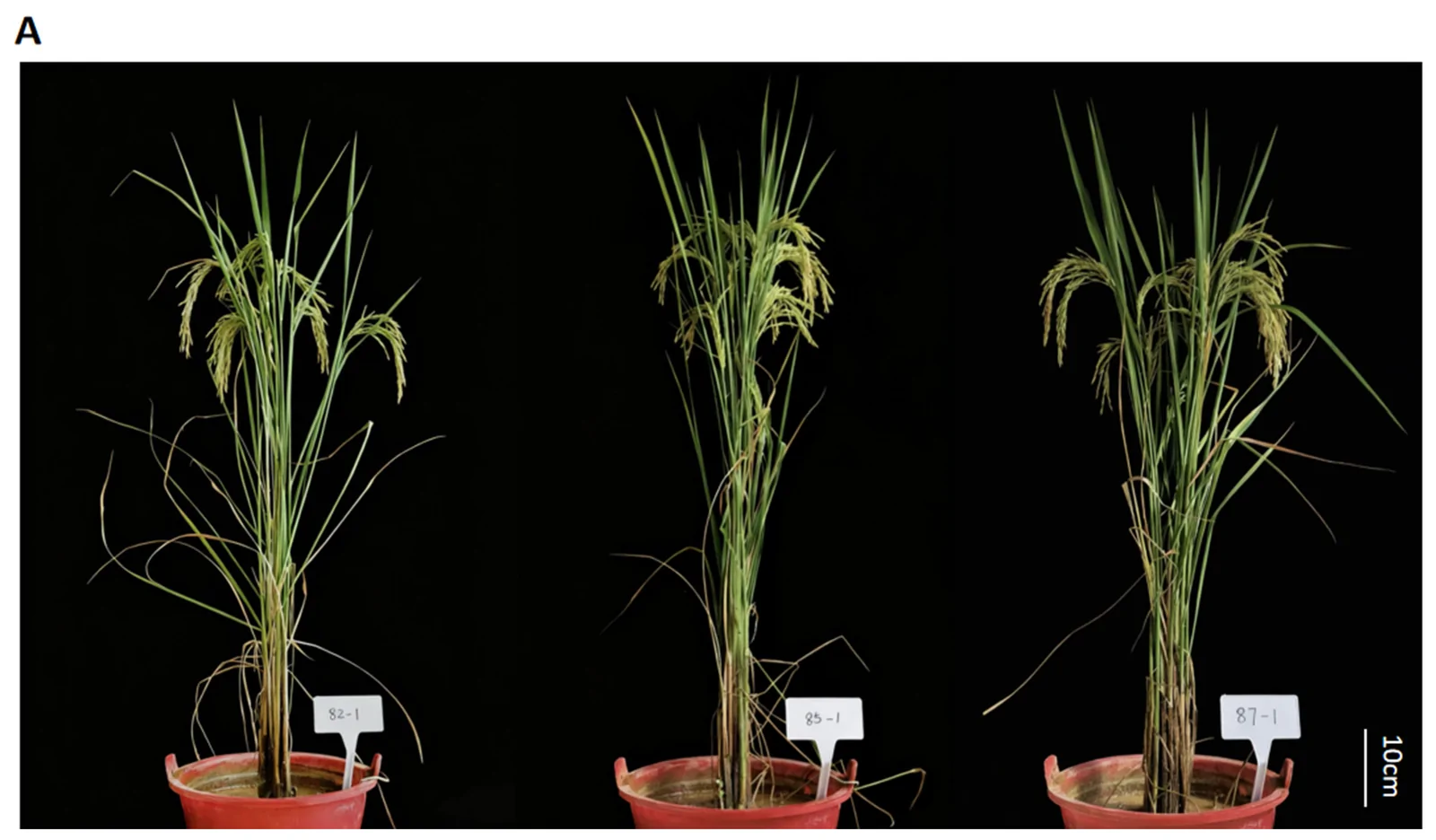

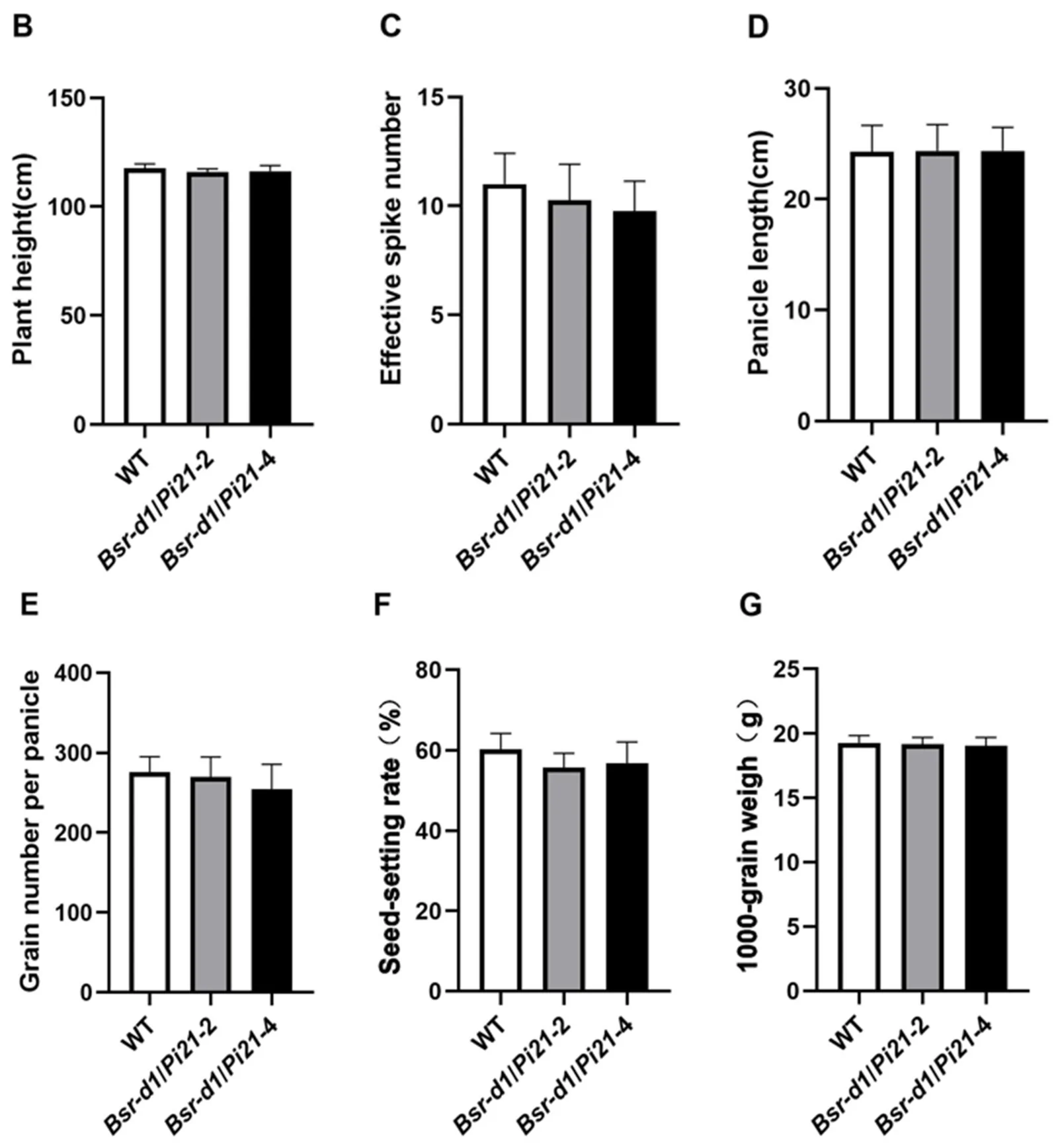
Comparison of major agronomic traits between wild-type Gengxiang B and *Bsr-d1*/*Pi21* double mutant lines. (**A**) Representative plant morphology. (**B**) Plant height (cm). (**C**) Number of effective panicles per plant. (**D**) Panicle length (cm). (**E**) Grain number per panicle. (**F**) Seed-setting rate (%). (**G**) 1000-grain weight (g). Data are mean ± SD (*n* = 8). Statistical significance was determined by one-way ANOVA followed by Dunnett’s test. No statistically detectable differences were found between the double mutants and the wild type (*p* > 0.05).

To investigate the effects of CRISPR/Cas9 editing of *Bsr-d1* and *Pi21* on agronomic traits, homozygous plants without a Cas9 background were selected from the T_2_ generation of double mutants and grown under identical cultivation conditions. No statistically detectable differences were observed between the *Bsr-d1*/*Pi21* double mutants and the wild type in plant height, number of effective panicles, panicle length, grains per panicle, seed-setting rate, or thousand-grain weight (*p* > 0.05, one-way ANOVA followed by Dunnett’s test) (Figure 5A–G, Table S3).

We further performed standardized grain quality testing on representative double mutant and wild-type materials, including brown rice length, grain transparency grade, amylose content, alkali spreading value, and gel consistency (Table 5). All grain quality parameters were comparable between the edited line and the original maintainer Gengxiang B. However, because the grain quality traits were measured on a single pooled sample per line due to limited grain availability, statistical analysis could not be performed for these traits. Collectively, these results demonstrate that CRISPR/Cas9-mediated knockout of *Bsr-d1* and *Pi21* improves blast resistance without producing statistically detectable differences in the assessed agronomic or grain quality traits under the tested field conditions.

## 3. Discussion

Modern rice breeding aims to integrate multiple desirable traits, balancing high yield, superior grain quality, and robust biotic stress resistance. Recent genome editing studies have successfully combined blast resistance with other valuable agronomic traits, including resistance to bacterial leaf blight, bacterial leaf streak, aroma, and cytoplasmic male sterility [21,22,26,27]. For instance, simultaneous editing of *Pi21* and *OsSULTR3;6* confers dual resistance to blast and bacterial leaf streak in a single genetic background [28]. Combined multi-gene transformation and multiplex editing pipelines have also delivered multi-resistant, high-yield rice varieties [22]. These advances demonstrate that CRISPR/Cas9 genome editing has become a transformative tool for blast resistance breeding, enabling rational design defined by multi-target manipulation and coordinated trait pyramiding. By integrating functional characterization of key susceptibility loci (e.g., *Pi21, Bsr-d1*), multiplex editing strategies, and defense pathway regulatory networks, elite rice varieties with durable blast resistance and superior agronomic performance can be rationally developed, thereby contributing to global food security.

In this study, we used CRISPR/Cas9 multiplex editing to simultaneously disrupt *Bsr-d1* and *Pi21*, generating novel blast-resistant rice germplasm for hybrid rice improvement. The recipient line Gengxiang B is an elite laboratory-preserved indica maintainer with outstanding field agronomic performance but extreme susceptibility to rice blast. Targeted editing of these two susceptibility genes successfully produced stable *Bsr-d1*/*Pi21* double homozygous mutants. Pathogen inoculation assays confirmed substantially enhanced leaf and panicle blast resistance compared with wild-type Gengxiang B. These results validate multiplex S gene editing as an effective strategy for rapidly improving blast resistance in elite rice maintainers.

Although progress has been made in single-gene editing, greater and more durable resistance is expected to be achieved through the simultaneous editing of multiple key targets with different mechanisms of action. This strategy has become an important trend in rice disease resistance breeding. For example, simultaneous editing of multiple negative regulatory genes such as *OsMads26* and *OsELF3-2* improved broad-spectrum resistance to multiple diseases in rice [29,30]. Studies have also shown that combining the major R gene *Pigm* with the S gene *Bsr-d1* successfully produced disease-resistant lines with both race-specific and broad-spectrum resistance [31]. These examples provide strong evidence for the synergistic improvement of rice blast resistance through multi-gene editing. In the present study, simultaneous editing of *Bsr-d1* and *Pi21* generated a double mutant line with significantly enhanced resistance. This line also showed no statistically detectable differences in major agronomic traits compared with the recipient material under field conditions and could therefore be directly used in our hybrid rice improvement program.

One limitation of this study is that off-target evaluation was based solely on in silico prediction using the CRISPR-GE tool. Although no high-risk off-target sites were predicted, experimental validation of the top-ranked candidate sites by Sanger sequencing was not performed. Therefore, potential off-target mutations cannot be completely ruled out. Future studies should include experimental validation to further confirm editing specificity. Additionally, the resistance phenotype observed here was based on a single *M. oryzae* isolate (H322); whether the enhanced resistance extends to other isolates remains to be determined.

Our rice breeding program has developed a series of edited rice materials targeting diverse agronomic traits via CRISPR/Cas9 technology to date, including optimized leaf morphology [32], early maturity [33], thermo-sensitive genic male sterility (TGMS) [34], photoperiod-thermo-sensitive genic male sterility (PTGMS) [35], glutinous endosperm [36], aromatic grain quality [33,37], altered grain shape [38], dual resistance to bacterial blight and bacterial leaf streak [28], and reduced cadmium accumulation [39]. Several of these edited elite lines have entered seed multiplication trials for hybrid rice commercialization. The successful development of blast-resistant *Bsr-d1/Pi21* double mutants in this study further expands our edited germplasm repository and reinforces the value of genome editing for accelerating elite rice varietal development.

## 4. Materials and Methods

### 4.1. Plant and Pathogen Materials

The recipient maintainer line Gengxiang B was co-developed by Guangxi University and Guangxi Lvhai Seed Co., Ltd., and serves as the nuclear maintainer for the cytoplasmic male sterile (CMS) line Gengxiang A. Multiple high-quality hybrid rice combinations derived from Gengxiang A have been widely cultivated across southern China. Gengxiang B exhibits superior grain quality and excellent general combining ability for hybrid rice production but is highly susceptible to rice blast. All rice plant materials were cultivated in experimental field plots managed by Guangxi University. The CRISPR/Cas9 binary vector system used in this research was kindly provided by Prof. Yaoguang Liu (South China Agricultural University). The *Magnaporthe oryzae* isolate H322 utilized for all inoculation assays was provided by Prof. Haowen Peng’s laboratory (Guangxi University).

### 4.2. Vector Construction and Rice Genetic Transformation

Target sites in *Bsr-d1* and *Pi21* were selected using the CRISPR-GE web tool [40]. The promoter–target fragment and the fragment containing the target sequence and single guide RNA (sgRNA) were amplified separately and then joined by overlapping PCR to generate the sgRNA expression cassettes. These cassettes were subsequently ligated into the CRISPR/Cas9 vector. After sequence verification, the correct CRISPR/Cas9 plasmid was introduced into *Agrobacterium tumefaciens* EHA105, and the resulting strain was used to transform Gengxiang B. Transgenic T_0_ plants were identified using the specific primers Cas9-F/Cas9-R and HPT-F/HPT-R. Target region fragments were amplified with *Bsr-d1*-TF/*Bsr-d1*-TR and *Pi21*-TF/*Pi21*-TR, and the amplified fragments from T_0_ and T_1_ plants were verified by Sanger sequencing. All primers were synthesized by Tsingke Biotechnology Co., Ltd. (Guangzhou, China).

The DSDecodeM online tool was used to analyze the Sanger sequencing results and determine target-site mutations [40]. Primers Cas9-F/Cas9-R and HPT-F/HPT-R were used to screen for transgene-free lines. Target sequences were amplified with specific primers, and the resulting products were sequenced by Sanger sequencing. The obtained sequences were compared with the reference sequences from the Rice Annotation Project Database (RAP-DB) to determine the edits in each gene.

### 4.3. Magnaporthe Oryzae Inoculation and Blast Phenotyping

In this experiment, wild-type Gengxiang B and mutant plants were inoculated with *M. oryzae* H322. For inoculum preparation, the preserved strains were transferred to potato dextrose agar (PDA) medium prepared in-house and cultured at 28 °C for 5 days. The mycelia were fragmented in sterile water, and the resulting mycelial suspension was spread onto fresh tomato oat medium prepared in-house. After the medium surface had air-dried, the plates were incubated at 28 °C in the dark for 3 days. Once aerial mycelia had formed, they were removed with a cotton swab, and the plates were washed with sterile water to remove residual mycelia. After air-drying, the plates were covered with two layers of sterile gauze and incubated at 28 °C under light for 3 days to induce conidiation. Before inoculation, rice seedlings at the three-leaf stage were selected, and a conidial suspension was prepared as described by He et al. [41]. The spore concentration was adjusted to 1 × 10^5^ spores/mL using a hemocytometer. The conidial suspension was sprayed evenly onto rice leaves, and the inoculated plants were covered with plastic wrap to maintain high humidity. The inoculated seedlings were kept in the dark for 3 days and then transferred to light for 5 days. Disease severity was then assessed as described by He et al. [41].

For quantitative disease evaluation, five representative leaves and five representative panicles per line were scored using a 0–9 severity scale. For leaf blast: 0 = no lesions, 1 = <5% leaf area affected, 3 = 5–25%, 5 = 26–50%, 7 = 51–75%, and 9 = >75% of leaf area affected. For panicle blast: 0 = no disease, 1 = disease on individual branches only, 3 = disease on the main axis or neck with minor yield loss, 5 = neck infection with partial grain filling, 7 = neck infection with severe yield loss, and 9 = almost total yield loss. The disease index (DI) was calculated as DI (%) = [Σ (disease grade × number of samples of that grade)/(total number of samples × 9)] × 100. Data are presented as mean ± SD (*n* = 5 leaves or panicle per line). Statistical analysis was performed using IBM SPSS Statistics for Windows, Version 26.0 (IBM Corp., Armonk, NY, USA). Homogeneity of variances was tested using Levene’s test. When variances were homogeneous (*p* > 0.05), one-way ANOVA followed by Dunnett’s multiple comparison test (with the wild type as the control) was used to compare the mutant lines with the wild type. *p* values < 0.05 were considered statistically significant.

### 4.4. Quantitative RT-PCR Gene Expression Analysis

Total RNA was extracted from fresh leaves collected at 0 and 24 h post-inoculation using RNAiso Easy reagent (TCH020, TaKaRa, Kyoto, Japan). After reverse transcription into cDNA, quantitative real-time PCR (qRT-PCR) was performed to analyze the expression of *Bsr-d1*, *Pi21* and defense-related genes in wild-type Gengxiang B and mutant plants. Three independent biological replicates were used for each sample, and each qRT-PCR reaction was performed in duplicate (technical replicates). Relative expression levels were calculated using the 2^−ΔΔCt^ method with *OsActin* as the internal control. Data are presented as mean ± SD (*n* = 3). Statistical analysis was performed using IBM SPSS Statistics for Windows, Version 26.0 (IBM Corp., Armonk, NY, USA). One-way ANOVA followed by Dunnett’s multiple comparison test (with the wild type as the control) was used to compare the double mutants with the wild type. *p* values < 0.05 were considered statistically significant.

### 4.5. Phenotypic Evaluation of Agronomic and Grain Quality Traits

Wild-type Gengxiang B and homozygous *Bsr-d1/Pi21* double mutant lines were planted in experimental field plots at Guangxi University. Each genotype was arranged in two rows with eight individual plants per row under standard local rice water and fertilizer management regimes. At physiological maturity, eight representative plants per line were randomly sampled to quantify plant height, effective panicle count, panicle length, grains per panicle, seed-setting rate, and 1000-grain weight (g). These eight plants are subsamples from a single field plot and should not be considered independent field replicates. Data are presented as mean ± SD (*n* = 8). Statistical analysis was performed using IBM SPSS Statistics for Windows, Version 26.0 (IBM Corp., Armonk, NY, USA). One-way ANOVA followed by Dunnett’s multiple comparison test (with the wild type as the control) was used to compare the double mutants with the wild type. *p* values < 0.05 were considered statistically significant.

Grain quality parameters were measured following official standard protocols, including brown rice length (mm), transparency grade, apparent amylose content (%), alkali spreading value grade, and gel consistency (mm). Due to limited grain availability, grain quality traits were measured on a single pooled sample per line; therefore, standard deviations could not be calculated and statistical analysis was not performed.

## Figures and Tables

**Table 1:** Genotypic frequencies of T_0_ transgenic plants.

No. of Plants	Target Site	Proportion of Genotype (%)			
		Wild Type	Heterozygous	Homozygous	Biallelic
15	*Bsr-d1*	6.67 (1)	20.00 (3)	73.33 (11)	53.33 (8)
	*Pi21*	33.33 (5)	6.67 (1)	60.00 (9)	53.33 (8)

**Table 2:** Distribution of mutation types at each target locus in T_0_ plants.

Target Locus	Frequence of Mutation Types (%)			
	WT	Insertion	Substitution	Deletion
*Bsr-d1*	6.67 (1)	46.67 (7)	6.67 (1)	40.00 (6)
*Pi21*	33.33 (5)	0.00 (0)	0.00 (0)	66.67 (10)

**Table 3:** Genome-wide off-target prediction for *Bsr-d1* and *Pi21* sgRNAs.

Target	Predicted Off-Target Sequence	Off-Target Score	Associated Gene ID	Genomic Region
*Bsr-d1*	GGCGAAGGGCATCTCGCAGA CGG	0.34	Null	Intergenic
	GGCGAAGGGCATCTCGCAGA CGG	0.34	OS03G0163300	CDS
	TGCAGGGCCTTCTCGCAGA CGG	0.277	Null	Intergenic
	GGGGAAGGCCTCCCCGAAGG GGG	0.169	OS02G0724600	CDS
*Pi21*	AGAAGCAGAGCTGGAAGATC TGG	0.177	OS11G0123500	CDS
	AGAACCTGAGAAAGAAGACC TGG	0.151	Null	Intergenic
	AGACGCTACGCAACAAGATC TGG	0.14	OS08G0241300	Intron

**Table 4:** Target-site allelic sequences and mutation types of tested materials.

Line Name	Gene	Target Site Sequence	Editing Types
Gengxiang B	*Bsr-d1*	GGCGAAGGCCTTCCCCCAGA	WT
	*Pi21*	AGAAGCTGTGCAAGAAGATC	WT
*Bsr-d1*/*Pi21*-2	*Bsr-d1*	GGCGAAGGCCTTCCCC--GA	−2 bp
	*Pi21*	AGAAGCTGTGCAAGAA--TC	−2 bp
*Bsr-d1*/*Pi21*-4	*Bsr-d1*	GGCGAAGGCCTTCCCCCGAGA	+1 bp
	*Pi21*	AGAAGCTG--------------	−14 bp

**Table 5:** Comparison of grain quality traits between the Bsr-d1/Pi21 double mutant line and the wild-type (Gengxiang B).

Name	Brown Rice Length (mm)	Transparency (Grade)	Amylose Content (%)	Alkali Spreading Value (Grade)	Gel Consistency (mm)
Gengxiang B	6.9	1.0	14.8	7.0	78.0
*Bsr-d1*/*Pi21*	6.9	1.0	14.8	7.0	76.0

Note: Values were obtained from a single pooled sample per line due to limited grain availability; therefore, standard deviations could not be calculated and statistical analysis was not performed.

## Data Availability

The original contributions presented in this study are included in the article and Supplementary Material. Raw phenotypic data, qRT-PCR Ct values, and Sanger sequencing chromatograms are provided as Supplementary Files (Tables S1–S3). Further inquiries can be directed to the corresponding author.

## Supplementary Materials

The following supporting information can be downloaded at: https://www.mdpi.com/article/10.3390/plants15172585/s1, Table S1: Relative expression levels (2^−ΔΔCt^) of target and defense-related genes in wild-type Gengxiang B and *Bsr-d1/Pi21* double mutants. Each value represents one biological replicate. OsActin was used as the internal control; Table S2: Rice blast disease severity scores (0–9) and disease index of wild-type Gengxiang B and *Bsr-d1/Pi21* double mutants. Table S3: Raw agronomic trait measurements of individual plants from wild-type Gengxiang B and *Bsr-d1/Pi21* double mutant lines. Eight plants were sampled per line as subsamples from a single field plot. Data are presented for descriptive comparison; statistical analysis was performed using one-way ANOVA followed by Dunnett’s test with the wild type as the control.
